# 

**Published:** 1878-07

**Authors:** 


### ARTICLE VIII.

### *Pennsylvania State Dental Society.*

The Executive Committee take pleasure in announcing the following programme for the meeting of this Society, at Bedford Springs, commencing July 30th, at 10 o'clock A. M., and continuing three days.

*Essays.*—1. Thirty years experience with Anæsthetics. By S. Welchens, D. D. S.

2. A review of the conservative treatment of the Dental Pulp. By Louis Jack, D. D. S.

3. Ante and Present Professional Education. By H. Gerhart, D. D. S.

4. The Accepted Creed. By Prof. C. N. Pierce, D. D. S.

5. Gold and Tin in combination. By Prof. D. D. Smith, D. D. S.

6. "Artificial Crowns." By Prof. E. T. Darby, D. D. S.

7. Why do fillings fail? The electro chemical theory of the "New Departure" *vs.* Defective Manipulation and Defective Structure. By W. H. Trueman, D. D. S.

8. "Tissues." By Marshall H. Webb, D. D. S.

9. Discoloration of the Teeth. By G. W. Adams, D. D. S.

10. Subject to be announced. By R. Huey, D. D. S.

11. Address of the retiring President. By C. S. Beck, D. D. S.

*Surgical Clinics.*—A tumor of the Inferior Maxillae extending from the first Molar on the left side, to the first Bicuspid on the right side, will be removed. By Prof. Chas. J. Essig, D. D. S.

*Operative Clinics.*—Cohesive gold impacted against and over frail walls of enamel, so that such walls may be encased and protected by gold, and restoration of the contour of missing tissue by the aid of a New Electro-Magnetic Mallet. By Marshall H. Webb, D. D. S.

The use of Williams' Cylinder illustrated. By Robert Henry, D. D. S.

Restoration of Contour by the aid of the Electro-Magnetic Mallet. By Geo. B. McDonneld, D. D. S.

Operation with the Electro-Magnetic Mallet. By E. P. Kremer, D. D. S.

Operation. By H. C. Longnecker, D. D. S.

A number of new appliances will be exhibited by inventors, including File Carriers, Syringes, Chairs, Engines and many others by manufacturers.

The committee desire to make this an interesting feature of the meeting, and will make suitable provision for the exhibition of any inventions or objects of interest to the society that may be presented.

Excursion tickets can be procured in New York, Baltimore, Washington, and in all the cities of this State; also at all the principal stations of the Philadelphia and Erie, the Northern Central and the Pennsylvania Rail Roads. Persons desiring to go from Bedford to the American Dental Association which meets at Niagara Falls the following Tuesday, can purchase tickets for Niagara Falls via Harrisburg or Tyrone, and at these points buy excursion tickets to Bedford, and as they return to these points resume their journey on their Niagara Falls' tickets.

Bedford Springs is one of the most healthful and popular summer resorts, consisting of a group of some 20 or 25 springs with names as various as their compositions, each spring being named after the predominating mineral held in solution.

The Hotel has ample accomodations for all, and the rates to persons attending the meeting will not exceed two dollars per day.

G. W. Klump,

Williamsport, Pa. *Chairman Executive Com.*

---

*American Dental Convention.*

The twenty-fourth annual meeting of the American Dental Convention, will be held at Niagara Falls, commencing August 6th, 1878. All members of the profession are invited to attend.

Ambler Tees,
*Secretary.*

### To Readers and Correspondents.

Communications intended for publication, and exchange journals and papers, should be addressed to the EDITOR OF THE AMERICAN JOURNAL OF DENTAL SCIENCE, 259 N. Eutaw St., Baltimore, Md. All letters relating to the business of the journal, or containing cash remittances, to be directed to Messrs. SNOWDEN & COWMAN. Articles for publication should be received by the editor at least three weeks previous to the first day of the month on which the number in which it is designed for them to appear, is issued.

ARTICLE VIII.—Pennsylvania State Dental Society.................... 130

ARTICLE IX —Cheiloplasty. By John H. Brinton M. D.................... 132

ARTICLE X.—Treatment of Stomatitis Materna. By E. P. Hurd, M. D.............. 134

ARTICLE XI.—Local Effects of Astringents upon the Blood Vessels. By Dr. A. Rosenstein.................... 135

### EDITORIAL, ETC.

Hope for the Dental Profession. The Cummings' Patent.................... 137

Chloroform and the Dentists.................... 140

College Changes.................... 143

### BIBLIOGRAPHICAL.

Proceedings of Southern and American Dental Conventions.................... 143

☞The American Journal of Dental Science will contain fifty pages of reading matter in each number, making a volume of about *Six Hundred* pages

EXCLUSIVE OF ADVERTISEMENTS

THE

### AMERICAN JOURNAL

OF

### DENTAL SCIENCE.

The *Journal* is issued on the first of each month and contains not less than *fifty* pages of reading matter in each number, exclusive of advertisements, making a volume of *six hundred* pages per annum.

In each number will be found Original Communications, Correspondence, Selected Articles, Monthly Summary, Bibliographical Notices and Editorial Matter, and every effort will be exerted to render the *Journal* worthy of the patronage of the Dental profession.

A generous co-operation is respectfully solicited from the members of the profession; and as the *Journal* has now a printing office of its own, which materially lessens the cost of its publication, it will be issued at the reduced subscription price of

**$2.50 Per Annum in Advance.**

Communications are respectfully solicited on all subjects pertaining to the practice of dentistry, as well as reports of cases occurring in dental practice.

### RATES OF ADVERTISING.

| | 1 MO. | 2 MO. | 3 MO. | 6 MO. | 12 MO. |
|---|---|---|---|---|---|
| 1 PAGE. | $15 00 | $22 50 | $30 00 | $50 00 | $100 00 |
| ½ " | 10 00 | 15 00 | 20 00 | 30 00 | 50 00 |
| ¼ " | 7 00 | 11 00 | 15 00 | 20 00 | 30 00 |
| ⅛ " | 5 00 | 8 00 | 11 00 | 15 00 | 20 00 |

All original communications designed for publication, works for review, new instruments for notice, exchanges, &c., should be directed to the editor, 259 N. Eutaw St., Baltimore, Md.

All advertisements and subscriptions should be addressed to

SNOWDEN & COWMAN,

*Publishers of the Am. Journal of Dental Science.*

86 W. FAYETTE ST. BET. CHARLES & LIBERTY STS.,

BALTIMORE, MD

